# Supplementary material for: A Muscle Load Feedback Application for Strength Training: A Proof-of-Concept Study
Source: Sports (Basel). 2023 Sep 5;11(9):170. doi: 10.3390/sports11090170 (PMC10534713; doi:10.3390/sports11090170)
Supplement: Supplementary file 1 [file sports-11-00170-s001.zip › Supplementary file S1.pdf]

Supplementary file S1: Primary and secondary muscle contributions

Machine	Primary muscle group(s)	Secondary muscle group(s)
Leg press	Quadriceps	Hamstrings
	Buttocks	Abductors
		Adductors
		Calves
Leg curl	Hamstrings	
Leg extension	Quadriceps	
Hip adduction	Adductors	
Hip abduction	Abductors	
Abdominal crunch	Abs	
	Obliques	
Torso rotation	Obliques	
Back extension	Low back	
Chest press	Chest	Shoulders anterior
		Triceps
Pectoral fly	Chest	
Rear deltoid/ Reverse pectoral fly	Upper back	
Seated row machine	Upper back	Lats
		Shoulders posterior
		Biceps
Low row horizontal cable	Upper back	Lats
		Shoulders posterior
		Biceps
Lat pulldown	Lats	Upper back
		Lower back
		Biceps
Triceps pushdown cable	Triceps	

Dual pulley pulldown seated	Lats	
	Biceps	
Pulley: biceps curl cable	Biceps	
Pulley: upright row 2pulleys	Shoulder anterior	Biceps
	Shoulder posterior	
