# Supplementary material for: A Muscle Load Feedback Application for Strength Training: A Proof-of-Concept Study
Source: Sports (Basel). 2023 Sep 5;11(9):170. doi: 10.3390/sports11090170 (PMC10534713; doi:10.3390/sports11090170)
Supplement: Supplementary file 1 [file sports-11-00170-s001.zip › Supplementary file S2.pdf]

Supplementary file S2: Muscle load estimation

To calculate the muscle load after each exercise, we use several assumptions

- The determined RM represents the weight for which 1 repetition leads to 100% muscle load.
- The relationship between repetitions and muscle load is linear and goes through (0,0), but is dependent on the used weight.
- 30 repetitions at 70% RM leads to a muscle load of 65% (this is set to the target load based on guidelines from the American College of Sports Medicine (16).
- The relationship between the weight and the maximum number of repetitions (corresponding to 100% muscle load is also linear.

These assumptions lead to the following equation:

$$Load = r * \frac{100}{(-1.5051*\left(\frac{100*w}{RM}\right)+151.5128} * \mathrm{mc}$$

Wherein:

- r is the number of repetitions done
- w is the used weight in kilograms
- RM is the previously determined 1 repetition max of the participant for the specific exercise
- mc is the muscle contribution for the specific exercise (for a primary muscle: mc=1; for a secondary muscle: mc=0.5).
