# Supplementary material for: A Muscle Load Feedback Application for Strength Training: A Proof-of-Concept Study
Source: Sports (Basel). 2023 Sep 5;11(9):170. doi: 10.3390/sports11090170 (PMC10534713; doi:10.3390/sports11090170)
Supplement: Supplementary file 1 [file sports-11-00170-s001.zip › Supplementary file S3.pdf]

Supplementary file S3: Muscle soreness questionnaire in Dutch (filled in as example)

Heeft u op dit moment spierpijn? *

- (●) Ja
- ( ) Nee

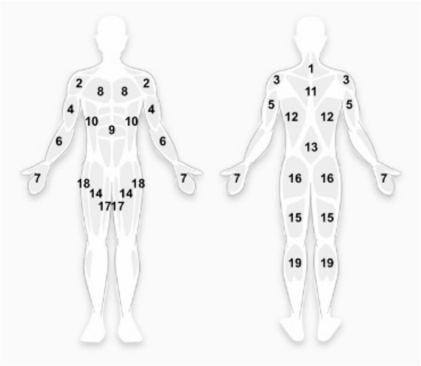

Geef aan waar u de afgelopen 7 dagen spierpijn heeft gehad. (Meerdere antwoorden mogelijk)

- [ ] 1. Nek
- [ ] 2. Schouders voorkant
- [ ] 3. Schouders achterkant
- [ ] 4. Biceps
- [x] 5. Triceps
- [ ] 6. Onderarmen
- [ ] 7. Handen
- [ ] 8. Borst
- [ ] 9. Rechte buikspieren
- [ ] 10. Schuine buikspieren
- [x] 11. Bovenrug
- [ ] 12. Zijkanten rug (lats)
- [ ] 13. Onderrug
- [ ] 14. Bovenbeen voorkant (quadriceps)
- [ ] 15. Bovenbeen achterkant (hamstrings)
- [ ] 16. Bilspieren
- [ ] 17. Bovenbeen binnenkant (lies)
- [ ] 18. Bovenbeen buitenkant
- [ ] 19. Onderbeen achterkant (kuit)

Wat was de intensiteit van de spierpijn in de achterkant van de bovenarmen (triceps) (5)?

Hele lichte spierpijn — Maximale spierpijn

Wat was de intensiteit van de spierpijn in de bovenrug (11)?

Hele lichte spierpijn — Maximale spierpijn
